# A Sliding-Gated Tactile Interface for Smartphone Side-Key Interaction

**DOI:** 10.3390/s26051436

**Published:** 2026-02-25

**Authors:** Fengyuan Yang, Wenqiang Yin, Chongxiang Pan, Jia Meng, Panpan Zhang, Xiong Pu

**Affiliations:** 1 Beijing Key Laboratory of High-Entropy Energy Materials and Devices, Beijing Institute of Nanoenergy and Nanosystems, Chinese Academy of Sciences, Beijing 101400, China; yangfengyuan@binn.cas.cn (F.Y.);; 2 School of Nanoscience and Engineering, University of Chinese Academy of Sciences, Beijing 100049, China; 3 Key Laboratory of Functional Materials and Devices for Informatics of Anhui Educational Institutions, School of Physics and Electronic Engineering, Fuyang Normal University, Fuyang 236037, China; 4 Ministry of Education Key Laboratory of Wooden Material Science and Application, Beijing Forestry University, Beijing 100083, China; 5 Center on Nanoenergy Research, School of Physical Science and Technology, Guangxi University, Nanning 530004, China

**Keywords:** sliding-gated tactile sensor, self-powered, smartphone side-key control, human–machine interaction

## Abstract

**Highlights:**

**What are the main findings?**

- A self-powered sliding-gated tactile interface is developed, generating direction-dependent voltage signals through triboelectrification-induced carrier redistribution.
- Dual-channel outputs provide stable features for machine-learning-based recognition of sliding and tapping gestures.
- The sensor enables discrimination of sliding direction, speed, pressure, and touch position without external power supply.

**What are the implications of the main findings?**

- The sensor provides a compact self-powered solution for multifunctional smartphone side-key interaction, including volume control, unlocking, and media operation.
- The proposed mechanism offers a practical route for intelligent tactile interfaces in human–machine interaction systems.

**Abstract:**

Achieving precise sliding perception is crucial for enhancing human–machine interactions. Despite the extensive investigation of tactile sensors for static pressure detection, they still face challenges in detecting dynamic information such as sliding direction, speed, pressure and position in interactive touch scenarios. Herein, we propose a self-powered tactile interface that realizes motion-to-electricity generation by electrostatically regulating the carrier concentration and transport in the semiconductive layer with a top gate in sliding movement. This tactile sliding interface can distinguish various dynamic mechanical information by generating voltage signals related to the sliding direction, speed, pressure, and touch position without external bias voltage. By combining machine-learning algorithms, electrical signals of six representative sliding-touch interactions were accurately classified with a recognition accuracy of 98.33%. Furthermore, by integrating sensors into the smartphone’s side button, customizable functions such as volume control, screen unlocking, and music switching were achieved. This work provides an innovative mechanism for sliding sensing in interactive electronic and intelligent control systems.

## 1. Introduction

Tactile perception is crucial for humans to interact with the physical world. Therefore, flexible tactile sensors have become indispensable components in next-generation human–machine interaction (HCI), wearable devices, and intelligent control systems [1,2,3,4,5]. These sensors can perceive external pressure and vibration stimuli and convert these physical signals into electrical outputs that electronic systems can readily process [6,7]. Depending on the transduction mechanism, flexible tactile pressure sensors are mainly divided into different types including piezoresistive [8,9,10], capacitive [11,12], piezoelectric [13,14], triboelectric [7,15,16] and flexoelectric [17,18]. Piezoresistive sensors have simple structures and high sensitivity; capacitive sensors provide rapid response and low power consumption, while triboelectric and piezoelectric devices enable self-powered operation without the use of external biasing [19,20,21,22]; flexoelectric sensors enable precise sensing of deflection and bending [18]. Most state-of-the-art pressure sensors are optimized for static or quasi-static force detection. However, interactive touch interfaces require sensing dynamic sliding behaviors, which involve motion direction, speed, contact pressure [23,24], etc. Traditional strategies to achieve sliding detection rely on large sensor arrays [25,26,27,28] or microstructured elastomer layers [29,30], requiring a large number of sensing units and signal channels, increased power demand, and difficulties in the production process. Moreover, the coupling of multiple mechanical parameters often leads to signal interference, reducing recognition accuracy [31,32]. Therefore, developing a low-power and directionally sensitive tactile interface capable of distinguishing sliding motions remains a major challenge for compact electronic systems.

In this work, we propose a self-powered sliding-gate-controlled tactile interface sensor that achieves motion-to-electricity generation by electrostatically regulating the carrier concentration and transport in the semiconductive layer with a top gate in sliding movement. “Self-powered” refers to the sensing interface itself, which generates electrical signals without any external bias applied to the sensor. In the device, a sliding object or finger acts as a moving gate electrified by the friction movement, which then dynamically adjusts the distribution of charge carriers and generates electrical signals along the sliding direction in an underneath polymer semiconductor through the electrostatic induction effect. This feature allows the device to distinguish different sliding directions, speeds, and pressures without external bias voltage. The system can accurately classify different signals by coupling dual-channel voltage outputs with machine-learning algorithms. The compact structural design facilitates integration into smartphone side keys, enabling intuitive gesture-based interaction on compact interfaces. This new type of sensor provides a scalable intelligent tactile solution for future human–machine interaction and interactive electronic devices.

## 2. Materials and Methods

### 2.1. Materials

The tactile interface sensor is made of a surface PTFE film (88 μm thick) and a glass substrate with a PEDOT: PSS layer spin coated underneath, as well as a gold electrode sputtered on the PEDOT: PSS layer (Figures S1 and S4). Before spin coating the PEDOT: PSS layer, the glass substrate was cleaned with ultrasound, acetone, ethanol, and deionized water. PEDOT: PSS solution was spin-coated at 1500 rpm and then annealed at 100 °C for 30 min to form a uniform conductive film with a thickness of about 18 μm. A patterned mask was used to deposit gold electrodes at both ends of the PEDOT: PSS layer by magnetron sputtering, and then external wires were connected for signal acquisition. The slider is an acrylic block with a 1 cm × 1 cm nylon film adhered to its lower surface. During the operation, the slider or finger moves on the PTFE surface, generating frictional charges, and the voltage output between the two electrodes is measured.

### 2.2. Electrical Measurement and Characterization

All electrical measurements were conducted at room temperature. The sliding motion is driven by the slider through a linear motor (P01-37 × 120F-HP, LinMot USA Inc., Lake Geneva, WI, USA). Contact separation and click gestures have been manually tested. The open circuit voltage (Voc) and short circuit current (Isc) were recorded using an electrostatic meter (Keithley 6517B, Keithley Instruments, Cleveland, OH, USA). An electrostatic voltmeter (Model 279, Monroe Electronics, Lyndonville, NY, USA) was used to measure the surface potential of PTFE and nylon films before and after friction. The surface morphology and thickness of the PEDOT: PSS film were characterized by SEM (Nova 8020, FEI Company, Hillsboro, OR, USA).

### 2.3. Hot-Point Probe Measurement for the Determination of Carrier Type

In order to study the regional changes in carrier types caused by frictional electrostatic modulation, the hot spot probe method was used. Two pairs of gold electrodes were deposited on the left, middle, and right regions of the PEDOT: PSS layer by magnetron sputtering using a patterned mask. Each electrode pair uses copper wires connected to an external circuit and heat source, and a heated soldering iron tip is used to locally raise the temperature of one electrode as a hot spot. The hot electrode is connected to the positive terminal of the multimeter (Fluke 117C True RMS multimeter, Fluke Corporation, Everett, WA, USA) to record the output voltage. The polarity of the measured voltage is used to determine the majority carrier type in the corresponding region. Measurements were taken before and after the PTFE slider interface contact to confirm the reversible region doping effect caused by the sliding-gate mechanism.

### 2.4. COMSOL Simulation

**Figure 1:**
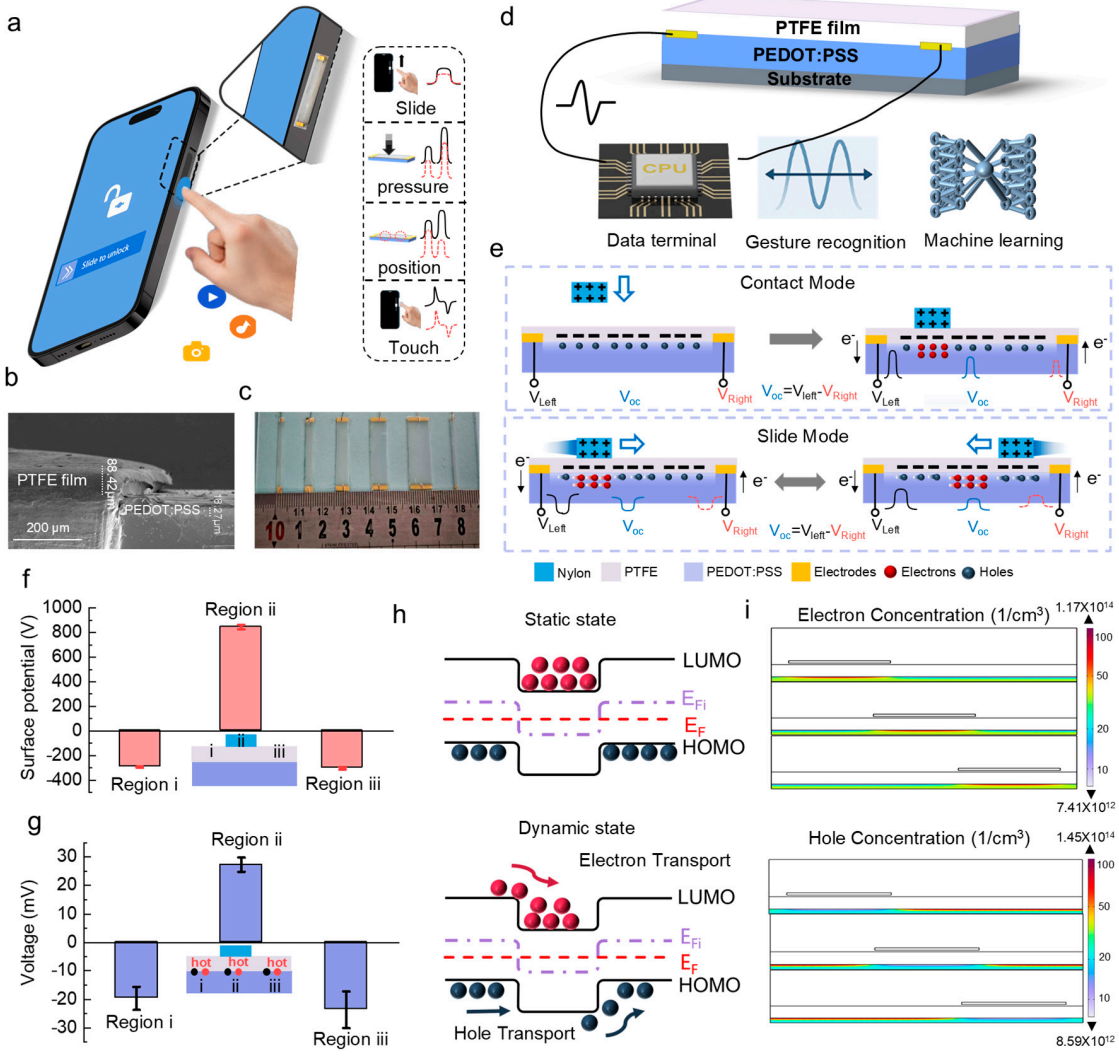
Sliding-gate-modulated tactile interface for smartphone side-key interaction and its operating mechanism. (**a**) Concept of the tactile interface sensor for smartphone side-key interaction. (**b**) Cross-sectional SEM image of the PTFE/PEDOT: PSS layered structure. (**c**) Photograph of the fabricated tactile interface sensors with different widths and dual-end electrode configuration. (**d**) Structure and signal processing flow of the tactile interface sensor. (**e**) Schematic illustration of charge carrier redistribution induced by contact mode and slide mode. (**f**) Surface potential distribution on the PTFE film after triboelectrification with the nylon slider. (**g**) Thermal voltage measured at different regions of the tactile interface (red and black dots denote the positive and negative measurement probes, respectively; the region color coding is consistent with (**e**)). (**h**) Schematic HOMO/LUMO-related energy-level landscape in PEDOT: PSS under sliding-gate electrostatic modulation. (**i**) Simulated spatial distributions of electron and hole concentrations in PEDOT: PSS under sliding-gate modulation.

Two-dimensional numerical simulations were carried out using COMSOL Multiphysics 6.2 with the Semiconductor Module to investigate the quasi-dynamic influence of triboelectric charges on electron and hole distributions. In the model, a charged triboelectric slider moves across an oppositely charged triboelectric film attached to a PEDOT: PSS semiconductive polymer layer, inducing electrostatic modulation of the underlying carriers. Since time-dependent environmental variations during sliding are difficult to explicitly account for, the sliding process was approximated by stationary solutions combined with a geometric sweep. The simulation domain was 22 μm × 90 μm in size. Surface charge densities of −10 and 30 μC m^−2^ were applied to the stationary film and the sliding object. The upper outer boundary of the simulation domain was defined as electrically neutral to ensure zero net charge at the boundary. Carrier distributions were evaluated by averaging the electron concentrations along two 1 μm long cutlines located at the upper left and upper right regions of the PEDOT: PSS layer. The simulation results are presented in Figure 1i and Figure S2.

## 3. Results and Discussion

### 3.1. The Concept and Mechanism of Our Interface Sensor

The proposed tactile interface sensor enables accurate, self-powered sensing of sliding and touching behaviors with discernible pressure and positional information. With these features, as shown in Figure 1a, it can be applied to smartphone side-key interaction, replacing traditional mechanical buttons to enhance functionality, sealing performance, and overall device integration. During operation, sliding, pressing, positioning, or touching gestures on the interface are converted into distinct electrical signals without an external power supply. Each gesture generates a characteristic electrical response. Sliding produces smooth and continuous signals that reflect the direction of movement; tapping produces a short-duration and localized pulse; pressure variations cause consistent changes in signal amplitude; different contact positions result in shifts in the voltage peak. These gesture-specific responses enable the sensor to convert subtle mechanical interactions into clear and interpretable electrical outputs.

Our previous work has demonstrated a sliding-gate strategy where the modulation of the population and transport of charge carriers in semiconductor materials by an electrostatic gate in sliding motion can result in electricity generation in a semiconductive channel [33]. The generated electrical signals are closely related to the sliding or touch motion of the moving gate. Therefore, based on this mechanism, we designed a sensor that adopts a typical sandwich structure as illustrated in Figure 1d. The top layer is a PTFE film placed above a glass-supported PEDOT: PSS layer. The glass substrate can also be replaced with other non-conductive supporting materials to better match the structural requirements of smartphone frames. A cross-sectional SEM image of the device is shown in Figure 1b, which shows the layered PTFE/PEDOT: PSS structure with intimate interfacial contact. A photograph of the fabricated sensors is shown in Figure 1c, which shows the elongated geometry, dual-end electrodes, and adjustable widths (2–12 mm) suitable for smartphone side-key integration. During operation, a nylon slider or the finger itself moves across the surface and functions as the sliding-gate, generating characteristic voltage signals between the two electrodes placed at the two ends of the PEDOT: PSS layer. These signals are collected by the MCU, processed through waveform recognition, and then used for machine-learning-based gesture classification.

Among the many conductive polymers and flexible semiconductor materials, we chose PEDOT: PSS as the semiconductive polymer layer. PEDOT: PSS is mainly used as the hole transport layer (HTL) in optoelectronic devices. PEDOT: PSS has good flexibility and processability. It can be used to prepare thin films on the substrate surface through methods such as spin coating, spraying, and printing.

The change in carrier concentration caused by friction can be quickly reflected as a voltage signal to improve the transient response ability of the signal. The conjugated structure of PEDOT enables continuous π-orbital overlap along the polymer backbone, allowing electrons to delocalize across multiple atomic sites (Figure S3). Upon oxidation, PEDOT loses electrons and forms positively charged carriers (holes), which are delocalized along the conjugated main chain and can migrate within the material. These hole carriers are readily modulated by external electrostatic fields, giving rise to the sliding-gate control effect. Additionally, the PEDOT: PSS layer forms a continuous and compact film with a well-defined interface to the PTFE layer. The sputtered Au electrodes exhibit compact contact on the PEDOT: PSS surface (Figure S4).

PTFE has strong electronegativity and tends to accumulate negative charges on its surface after sliding friction. Human fingers or nylon sliders are more likely to accumulate positive charges on their surfaces after sliding friction. As the total quantity of the tribo-positive and tribo-negative charges is equal, the electrostatic charge density in the top slider should be much higher than that in the PTFE film, considering its smaller area. Therefore, for the area not covered by the slider, the electrostatic electric field should be dominated by the negative space charges in PTFE film, while, for the area covered by the slider, the electric field is dominated by the net tribo-positive charges in the slider. PEDOT: PSS is a typical p-type semiconductive polymer (Figure 1h). It should be noted that charge transport in PEDOT: PSS is dominated by polaron/bipolaron hopping in a disordered polymer network. Therefore, the diagram is only used as an intuitive schematic for electrostatically induced energy-level shift and carrier redistribution, rather than a crystalline-semiconductor band structure. In the contact area, the negative charges on the PTFE surface are close to the PEDOT: PSS interface, generating a downward electrostatic field at the interface. This electric field reduces the concentration of dominant charge carriers (holes) in PEDOT: PSS, resulting in a locally hole-depleted region (reduced hole-dominated transport). Hole concentration occurred, forming a hole-enriched region. Under dynamic conditions, the positions of the “de-doped” region and the “enhanced p-type region” change depending on the position of the nylon slider above (Figure 1e,i).

When the nylon slider moves forward from left to right, the PEDOT: PSS in the trailing area is mainly affected by the electric field formed by the negative charge on PTFE, causing electrons to drift forward. Meanwhile, in the front (non-contact) “p-type enhanced region”, the moving positively charged slider will repel the holes forward. As a result, the overall carrier distribution moves in the sliding direction, and a potential gradient pointing toward the sliding direction is formed within the PEDOT: PSS layer. In short-circuit conditions, electrons flow from the left-end electrode to the right-end electrode through an external circuit to balance the charge. As long as the slider continues to move, the current generation continues. If the sliding direction is reversed, the current direction also reverses, which is consistent with the experimental observations. To verify this interfacial modulation mechanism, we first examined the electrostatic surface potential distribution generated by frictional charging between the PTFE film and the nylon slider (Figure 1f). As the slider moves back and forth, the accumulated charges increase gradually, and the final surface potential exhibits opposite polarities in different regions depending on the resting position of the slider. The surface potentials on the non-overlapping regions were −295 V and −304 V, while the surface potential on the overlapping region was +845 V, confirming the above reasoning that the regions with and without the slider exhibit opposite net electrostatic charges.

To experimentally confirm that the regions with and without the slider will induce a variation in the carrier population differently in the underneath PEDOT: PSS layer, we measured the diffusion voltage of thermally generated carriers to determine the carrier type in different areas of the PEDOT: PSS surface. In all measurements, the positive terminal of the multimeter was connected to the hot probe. When p-type PEDOT: PSS is heated using a hot spot probe, the contact area is electron-dominated transport, and the hot spot probe causes electrons to diffuse away, resulting in a positive non-equilibrium thermoelectric potential. When the non-contact area is heated, the thermal probe causes the holes to diffuse away, resulting in a negative non-equilibrium thermoelectric potential (Figure 1g). Furthermore, we simulated the electron concentration and hole concentration through COMSOL Multiphysics, which resulted in consistent observations with the above discussion (Figure 1i and Figure S3). The sliding-gate-induced electrostatic field leads to electron concentrations and hole concentrations in the different PEDOT: PSS layer regions. This simulated carrier redistribution supports the proposed sliding-gate modulation mechanism.

### 3.2. The Performance of Tactile Interface Sensor

**Figure 2:**
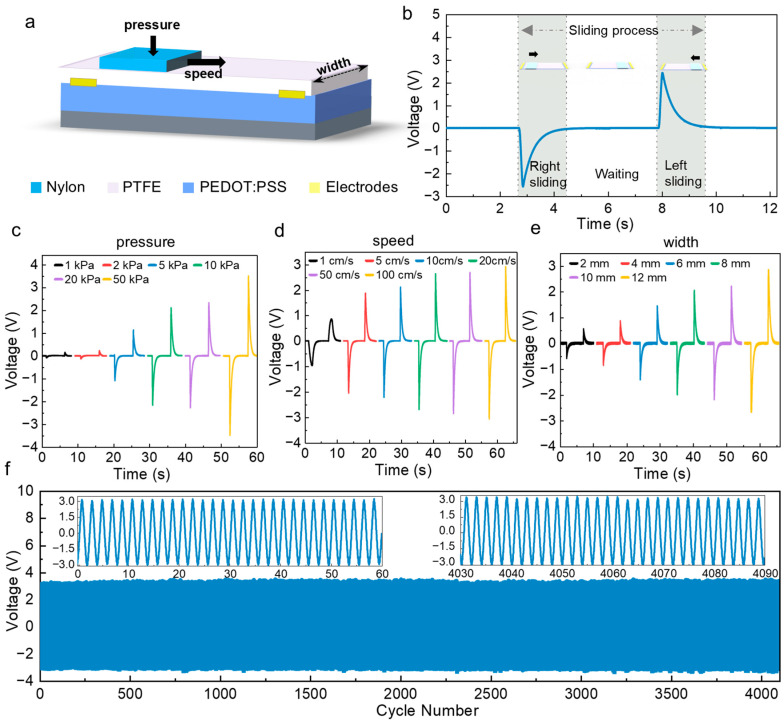
Electrical performance of the sliding-gate-controlled tactile interface sensor. (**a**) Experimental setup for sliding tests using a nylon slider. (**b**) Representative voltage output under reciprocating sliding motion. (**c**) Voltage output under different pressures. (**d**) Voltage output under different sliding speeds. (**e**) Voltage output for sensors with different effective widths. (**f**) Output stability of the sensor under repeated sliding cycles.

To further understand the sliding perception capabilities of our tactile interface sensor, we investigated the effects of sliding speed, normal pressure, and sensor width on the electrical output using a nylon slider (Figure 2a). In these tests, the sliding motion was reciprocating, with a 5 s pause after each cycle (Figure 2b). Unless otherwise specified, the base conditions were set to a speed of 10 cm/s, a normal pressure of 20 kPa, and a sensor width of 12 mm. On this basis, the open-circuit voltage and short-circuit current show significant responses to the changes in sliding speed, pressure and width (Figure 2c and Figure S4).

As shown in Figure 2c, as the applied pressure increases from 1 kPa to 50 kPa, the output voltage rises from 0.14 V to 3.46 V, indicating that there is a positive correlation between the contact force and the output signal amplitude. Especially between 1 kPa and 2 kPa, the output changes significantly, indicating that there may be an initial threshold of effective contact; when the pressure exceeds 50 kPa, the output tends to be saturated, and the charge accumulation may not increase significantly due to the saturation of the contact area. This pressure response can be used to identify the contact pressure within a reasonable range in practical applications and to suppress signal interference induced by accidental contact or dropping the device.

PEDOT: PSS may also exhibit a piezoresistive response under compression; however, the output voltage in this work was measured under near-open-circuit conditions using an electrometer with ultra-high input impedance. A control resistance measurement under a constant current of 1 µA (voltage compliance > 210 V) shows only a slight resistance variation in the GΩ range from 0 to 50 kPa (Figure S11a), indicating that piezoresistive modulation is not the dominant origin of the pressure-dependent voltage amplitude (Figure S11b).

Figure 2d shows that as the sliding speed increases from 1 cm/s to 100 cm/s, the output voltage amplitude continues to rise. This phenomenon reflects that the higher the sliding speed, the faster the friction charge moves at the interface, thus enhancing the electrostatic control effect. Considering that sensors with different widths can be designed according to the size of mobile phones in practice, we further studied the influence of width on electrical output (Figure 2e). The results show that when the width of the sensor increases from 2 mm to 12 mm, the output voltage increases accordingly, which may be due to the greater effective contact area leading to stronger charge accumulation. These results indicate that the sensor output varies systematically with sliding speed, applied pressure, and contact width. In order to verify the durability of the device, the sensor maintains a stable output waveform in 4000 cycles without significant signal attenuation, showing excellent operational stability and mechanical reliability.

### 3.3. The Signals of Different Gestures by 2-Electrode Measurement

**Figure 3:**
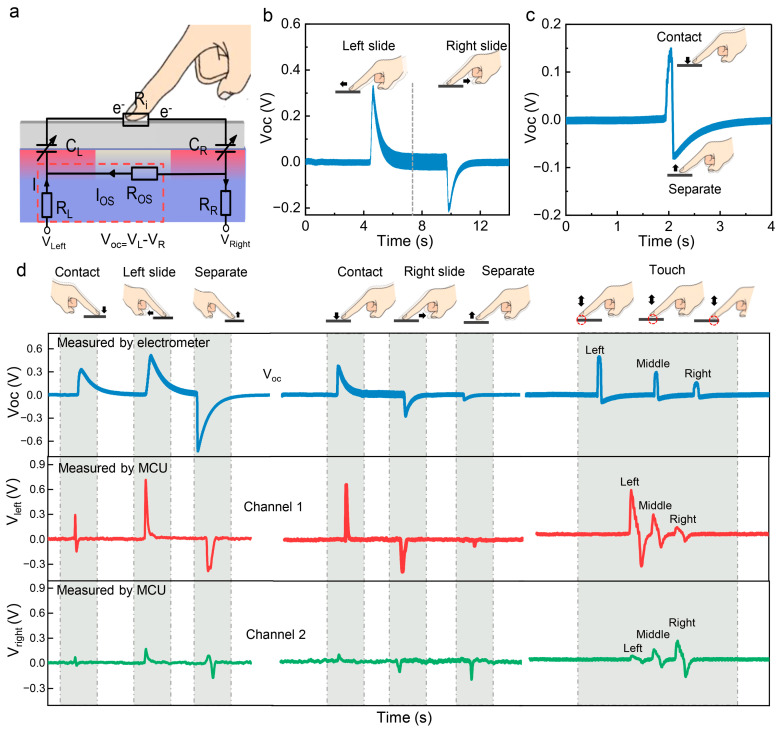
The signals of 2-electrode intelligent controller. (**a**) Equivalent circuit model of the sensor. (**b**) The open circuit voltage outputs under left and right sliding events. (**c**) The open circuit voltage signals during contact and separation events. (**d**) The voltage signals measured by the electrometer and MCU, including contact–slide–separation and position-dependent touch.

In order to systematically understand the voltage response law of our tactile interface sensor under sliding and touch behaviors, we constructed an equivalent circuit model corresponding to Figure 3a. This model includes two interface capacitors C_L_ and C_R_, located between the PTFE layer at the left and right ends of the device and the PEDOT: PSS ion conductive layer. At the same time, equivalent resistances R_L_, R_R_, and R_os_ corresponding to the enhanced P-type region and the undoped region are introduced, which are related to the carrier transport process in the PEDOT: PSS layer. The output of the device is determined by the interface capacitors (C_L_, C_R_) and the equivalent resistances of each region (R_L_, R_R_, R_os_). The change in capacitance promotes the establishment of potential, and the change in resistance reflects carrier migration. The two work together to affect the amplitude and polarity of the output waveform.

During the sliding process, the frictional charge on the PTFE–finger interface regulates the regional potential of PEDOT: PSS, leading to local changes in its band structure and carrier concentration, resulting in dynamic changes in C_L_, C_R_, R_L_, and R_OS_. The position of the slider determines whether the potential distribution on the left and right interfaces is symmetrical, and the device thus forms an asymmetric capacitance–resistance network controlled by position.

When a finger touches the left area of the device, the downward electrostatic field causes a decrease in the concentration of major charge carriers (holes) in the PEDOT: PSS layer, resulting in an increase in the equivalent resistance R_L_. Meanwhile, the change in interface potential leads to significant changes in C_L_. The interference of PEDOT: PSS on the right side is relatively weak, so C_R_ and R_R_ remain basically unchanged. In this asymmetric state, the output voltage of the device exhibits distinct polarity characteristics. When the slider slides to the right, the direction of the region control reverses, causing the polarity of the waveform to reverse accordingly. This polarity switching phenomenon is a key feature of sliding direction recognition and an important advantage of this device compared to traditional resistive/capacitive touch schemes.

To compare with traditional triboelectric nanogenerators (TENGs), we fabricated a TENG with the same mechanical structure as our device, but without the PEDOT: PSS semiconductive layer. The proposed tactile sensor introduces additional electrostatic regulation and carrier transport mechanisms in its equivalent circuit. In conventional TENGs, the output is mainly governed by the interface capacitances (such as C_L_ and C_R_) together with a fixed internal resistance R (Figure S7). Its response mechanism is based on electrostatic induction and does not involve the dynamic modulation of internal charge carriers. Our device introduces position-dependent adjustable resistance elements R_L_ and R_R_, as well as R_os_ controlled by carrier transport, to construct an asymmetric RC network sensitive to sliding position, which can achieve directional voltage output.

Figure 3b,c shows the output V_oc_ of the device under basic gestures, such as left slide, right slide, contact, and separation. Left and right sliding produce opposite signal polarities, with amplitudes around 0.4 V. This confirms the strong direction sensitivity. The response depends on the sliding speed and applied pressure. Light tapping generates short bipolar spikes, which come from sudden changes in interface capacitance during contact or release.

To study the practical performance, we measured the waveforms under compound gestures. These include contact-slide-release in different directions and repeated taps at different positions. A high-impedance electrometer and a dual-channel MCU ADC were used for measuring V_oc_ and V_left_/V_right_, respectively (Figure 3d). For the sequence of contact-left slide-release, the electrometer shows a positive pulse on contact, a second positive signal during sliding, and a negative pulse at separation. Reversing the gesture changes the polarity of the sliding and release signals, but the contact pulse remains the same. These results match the single gesture waveforms in Figure 3b,c. When tapping at multiple lateral positions, the signal amplitude decreases steadily from left to right.

The blue curve of V_oc_ in Figure 3d is from the electrometer. The electrometer has a high input impedance (above 10^14^ Ω), which allows the accurate tracking of charge buildup and decay without disturbing the interface. After sliding, the residual charges on the PTFE surface decay slowly over several seconds. This behavior may result from air ionization, surface leakage, carrier redistribution in the PEDOT: PSS layer, or the slow discharge of interfacial capacitance. This creates a relaxation tail in the waveform.

The red (CH1, V_left_) and green (CH2, V_right_) curves show signals measured by the MCU. Its lower input impedance (10^4^–10^5^ Ω) creates a discharge path, so charge decay is faster. As a result, the relaxation tail is shorter. In the MCU waveforms, contact and separation appear as sharp, narrow pulses. Sliding produces broader signals. The polarity reflects the direction: left sliding gives a positive peak in CH1 and CH2, while right sliding reverses them.

For touching applied at different lateral positions, the signal amplitude depends on the proximity to each electrode. When the contact is closer to the CH1 side, CH1 produces the dominant response, whereas CH2 dominates as the contact shifts toward the opposite side. This position-dependent amplitude distribution indicates that the electrical response of the device is spatially correlated with the tapping location.

A first-order IIR low-pass filter was applied at the MCU input. This reduces high-frequency noise and smooths the waveform. It also slightly lowers peak amplitude and introduces small delays at signal edges. The filter helps suppress environmental noise and fingertip-induced drift. It also improves signal contrast. The electrometer captures the intrinsic relaxation behavior of friction-induced charges, whereas the MCU reflects the device response under practical operating conditions. Despite the different measurement configurations, both measurements exhibit consistent signal trends, supporting the feasibility of the sliding-gate-controlled carrier modulation mechanism for directional sensing and multimodal interaction.

### 3.4. Machine-Learning-Assisted Gesture Recognition and Interactive Control

**Figure 4:**
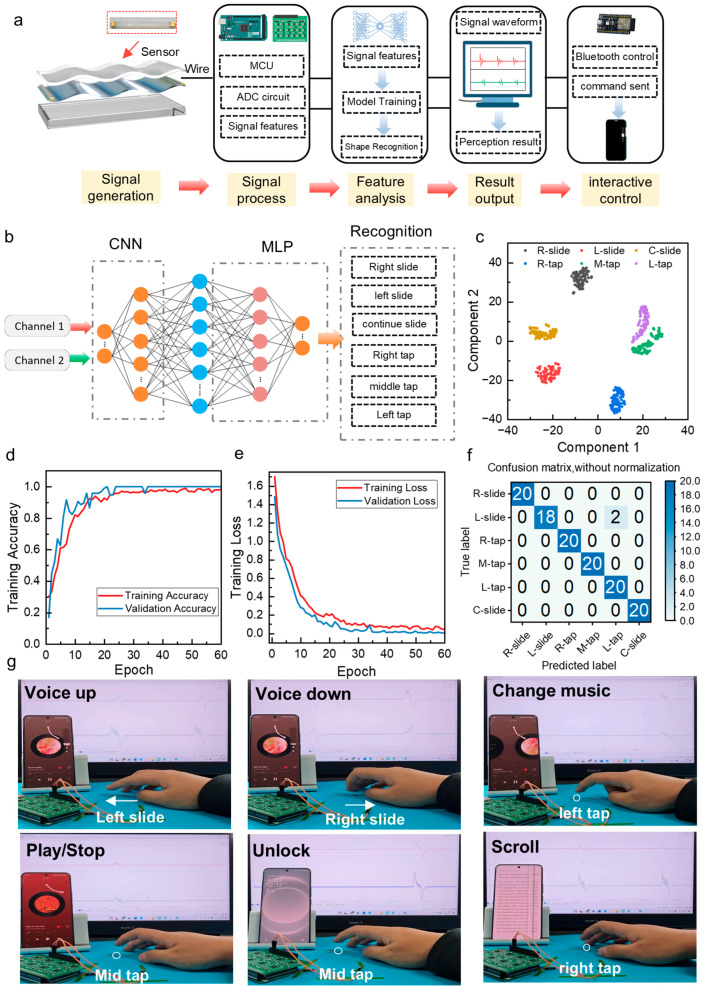
Dual-channel gesture recognition of the tactile interface sensor. (**a**) Signal processing and recognition procession from self-powered tactile sensing to wireless interactive control. (**b**) Machine-learning model for gesture classification using dual-channel inputs. (**c**) T-SNE visualization of dual-channel gesture features. (**d**) Training and validation accuracy during model learning. (**e**) Training and validation loss curves. (**f**) Confusion matrix for six representative gestures. (**g**) Gesture-controlled functions including volume adjustment, media switching, play/pause, screen unlocking, and page scrolling.

We implemented a complete signal acquisition and classification process to enable real-time gesture recognition, as shown in Figure 4a. When a user performs sliding or tapping gestures, the tactile interface sensor converts the mechanical input into self-powered electrical signals. These signals are first conditioned through a dedicated analog front-end circuit that performs amplification and low-pass filtering to remove ambient noise and baseline drift. The filtered dual-channel waveforms are then captured by the ADCs of an MCU and transmitted to a host computer for machine-learning–based gesture classification. Based on the recognition results, the control of the phone is achieved through a dedicated Bluetooth chip, achieving human–machine interaction.

Six representative gestures were selected as classification targets: left slide, right slide, continuous slide, left tap, right tap, and middle tap. Each gesture produces a distinct spatiotemporal waveform pair across CH1 and CH2, serving as input features. Using deep-learning algorithms to classify and recognize dynamic gesture signals, deep-learning models can be built with convolutional neural networks (CNNs). A CNN extracts features from the dynamic tactile signals. It then fuses these features and performs the final classification (Figure 4b). Usually, 80% of the data is randomly selected as training data, and the remaining 20% is used as testing data. During the training process, samples are extracted from the dataset, and the network is updated at each training step. The waveform features extracted from different gestures were projected into a two-dimensional space using t-distributed stochastic neighbor embedding (t-SNE) in Figure 4c. The resulting distribution shows that samples from the same gesture type cluster closely, while different gesture classes remain clearly separated. In a sequence of 60 training steps, the accuracy of training and testing continues to improve, while the loss gradually decreases. As shown in Figure 4d,e, both the training and validation accuracies increase rapidly with the epoch and gradually converge, indicating stable model optimization without overfitting. The confusion matrix in Figure 4f further verifies the classification performance, with all six gesture types correctly recognized and no misclassification observed in the test set. These results indicate that the dual-channel signals provide stable and discriminative features for gesture recognition.

To evaluate the practical applicability of the tactile interface sensor, we integrated the device into the side keys of commercial smartphones, as illustrated in Figure 4a. In this configuration, the sensor operates without an external power supply and supports multiple gesture-based interaction modes. Different gestures are identified according to their waveform features and subsequently assigned to corresponding control functions.

During the demonstration, vertical sliding motions were used to perform functions typically achieved by mechanical buttons, such as system volume adjustment and page switching (Figure 4g and Movie S1). Repeated touch events at a designated position were used to trigger screen unlocking. Beyond its multimodal interaction capability, the sensor also exhibits stable performance under practical usage conditions, indicating good environmental reliability. The proposed sensor features a fully sealed architecture without moving components. Traditional mechanical buttons depend on physical deformation and are susceptible to wear and structural gaps. The PTFE surface exhibits intrinsic hydrophobicity, with a static water contact angle above 90° (Figure S9), which provides inherent resistance to moisture and splashing. This structural sealing and surface wettability enhance operational reliability in daily use and moderately harsh environments and facilitate integration into compact electronic enclosures.

## 4. Conclusions

In summary, we have developed a sliding-gate-controlled tactile interface sensor for self-powered gesture recognition and smartphone side-key applications. The device relies on electrostatic modulation induced by triboelectrification. This modulation changes the local carrier distribution in the semiconductive PEDOT: PSS thin film. The carrier distribution shifts during sliding or tapping. As a result, the output voltage depends on the sliding direction. The sensor exhibits sensitivity to sliding speed, pressure, and contact position, with robust outputs over extended periods. These outputs serve as input features for machine-learning classification, achieving a recognition accuracy of 98.33% for six different gestures on the test set. In addition, we demonstrated the control of the smartphone using the sensor, achieving customizable features such as slide-based unlocking, music control, and page scrolling. This work provides a scalable electrostatic control touch solution for the control of smart devices, offering a new approach for future intelligent human–machine interaction systems.

## Data Availability

All relevant data that support the results of this study are available within the article and its Supplementary Materials. Further data are available from the corresponding authors upon reasonable request.

## Supplementary Materials

The following supporting information can be downloaded at: https://www.mdpi.com/article/10.3390/s26051436/s1, Figure S1: Structural diagram and photo of the sensor. Figure S2: Simulated surface potential under sliding-gate modulation. Figure S3: Chemical structure of PEDOT: PSS. Figure S4: Scanning electron microscopy images of the PEDOT: PSS layer and electrode interface. Figure S5: Open-circuit voltage and short-circuit current of the tactile interface sensor measured by an electrometer under varied sliding conditions. Figure S6: Voltage outputs of the tactile interface sensor measured by an MCU under varying mechanical conditions (baseline not zeroed). Figure S7: Comparison of equivalent circuit models and electrical outputs between the proposed sliding-gate tactile sensor and a conventional triboelectric nanogenerator. Figure S8: Voltage signals measured by different readout circuits under tapping and sliding gestures. Figure S9: Contact angle of water on PTFE. Figure S10: Representative waveform data of six gesture classes. Figure S11: Pressure-dependent resistance variation and corresponding voltage outputs of the sliding-gated tactile interface sensor. Figure S12: (a) Output under different temperatures; (b) output under different relative humidities. Figure S13: Output before and after cleaning with alcohol wipes. Table S1: Performance comparison of representative tactile/gesture sensors in terms of power supply, channel configuration, recognition mode, durability, and integration level. Table S2: Comparison between the proposed sliding-gated tactile interface and representative capacitive side-key solutions used in smartphones. Table S3: Layer-wise configuration of the CNN + MLP network used for gesture classification. Movie S1: Gesture-controlled functions including volume adjustment, media switching, play/pause, screen unlocking, and page scrolling. References [6,33,34,35,36,37,38,39,40,41,42] are cited in the Supplementary Materials.
