# Supplementary material for: A Sliding-Gated Tactile Interface for Smartphone Side-Key Interaction
Source: Sensors (Basel). 2026 Feb 25;26(5):1436. doi: 10.3390/s26051436 (PMC12987069; doi:10.3390/s26051436)
Supplement: Supplementary file 1 [file sensors-26-01436-s001.zip › Supporting Information.pdf]

Supporting Information

A Sliding-Gated Tactile Interface for Smartphone Side-Key Interaction

Fengyuan Yang [1,2], Wenqiang Yin [1,2], Chongxiang Pan [3], Jia Meng [1], Panpan Zhang [4,*] and Xiong Pu [1,2,5,*]

[1] Beijing Key Laboratory of High-Entropy Energy Materials and Devices, Beijing Institute of Nanoenergy and Nanosystems, Chinese Academy of Sciences, Beijing 101400, China
[2] School of Nanoscience and Engineering, University of Chinese Academy of Sciences, Beijing 100049, China
[3] Key Laboratory of Functional Materials and Devices for Informatics of Anhui Educational Institutions, School of Physics and Electronic Engineering, Fuyang Normal University, Fuyang 236037, China
[4] Ministry of Education Key Laboratory of Wooden Material Science and Application, Beijing Forestry University, Beijing 100083, China
[5] Center on Nanoenergy Research, School of Physical Science and Technology, Guangxi University, Nanning 530004, China
* Correspondence: zhangpanpan2024@bjfu.edu.cn (P.Z.); puxiong@binn.cas.cn (X.P.)

Supplementary Note S1

Our previous work has demonstrated a sliding-gate strategy where the modulation of the population and transport of charge carriers in semiconductor materials by an electrostatic gate in sliding motion can result in electricity generation in a semiconductive channel[1]. To provide an intuitive semiconductor-type equivalent-circuit interpretation, we refer to our previous sliding-gate FENG framework. Previous work summarized the relationship between the external electrostatic field/surface potential and the induced carrier density. It also provides the resulting equivalent circuit and the derived Voc/Isc expressions. We cite these results here to clarify the physical meaning of the circuit elements used in this work (**Figure S7**).

Note that, unlike crystalline Si, PEDOT:PSS is a disordered conducting polymer with polaron/bipolaron hopping transport. Therefore, the framework is used as an effective, phenomenological analogy to describe electrostatic modulation and circuit behavior. It is not intended as a rigorous band-structure treatment.

The short-circuit current can be expressed by:

$$I_{SC} = wv\frac{L}{l}\frac{\varepsilon}{d}V_{surf} \tag{S1}$$

The open-circuit voltage can be expressed by:

$$V_{oc} = wv\frac{L}{l}R\frac{\varepsilon}{d}V_{surf} \tag{S2}$$

where w and l are the width and length (the sliding is along the length direction, and the width is perpendicular to the sliding direction) of the nylon slider. L is the length of the PTFE film. ε is the permittivity of the PTFE film. v is the speed of the slider. d is the thickness of the PTFE film, and V_{surf} is the surface potential of the PTFE film. R is the resistance of the PEDOT:PSS, $R = R_{os} + R_R + R_L$. R_{os} is the resistance of the overlapped area of PEDOT:PSS with the top slider. $R_R + R_L$ is the sum of the contact resistance and the resistance of the non-overlapped area of PEDOT:PSS with the slider. R_i is the imaginary resistance of the slider. C_L and C_R are the capacitors formed by the left and right non-overlapped regions.

Equations S1 and S2 describe the quasi-static outputs of the sliding-gate model. The transient waveform and relaxation behavior are governed by the RC dynamics of the equivalent circuit.

From Equation S2, R represents the effective transport/leakage pathways in the conductive channel. A higher resistance slows charge redistribution and leakage, allowing the electrostatic potential to build up more easily under quasi-static conditions. In our device, the measured resistance changes only slightly with pressure (**Figure S11**a). This suggests that the pressure-dependent voltage amplitude is mainly determined by electrostatic/capacitive effects rather than by resistance modulation.

The relaxation of the output voltage after a transient event can be approximated by a first-order RC response. Accordingly, the voltage decay can be written as:

$$V(t) = V_0 \exp\left(-\frac{t}{\tau}\right) \quad \text{(S3)}$$

$$\tau \approx \mathrm{R}_{eff}\, C_{eff} \quad \text{(S4)}$$

where V_0 is the initial voltage at the beginning of the relaxation process, R_{eff} is the effective resistance, C_{eff} is the effective capacitance. t is time, and τ is the relaxation time constant. Here, τ characterizes how fast the signal returns to its baseline, and it is primarily determined by the effective resistance and capacitance in the equivalent circuit.

Supplementary Figures

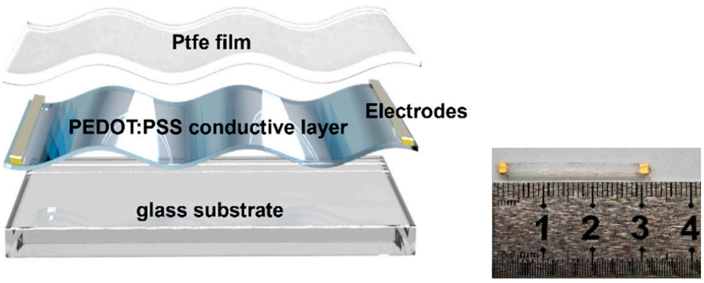

Figure S1. Structural diagram and photo of the sensor.

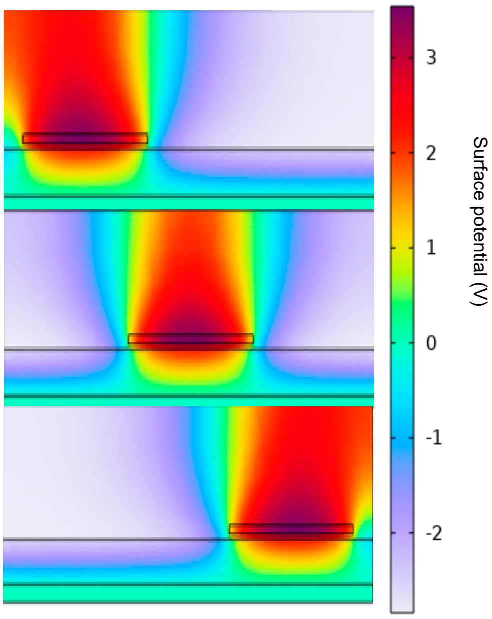

Figure S2. Simulated surface potential under sliding-gate modulation.

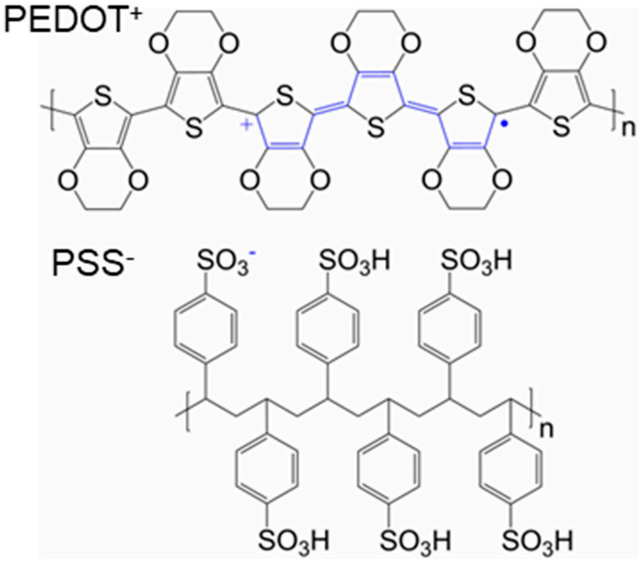

Figure S3. Chemical structure of PEDOT:PSS.

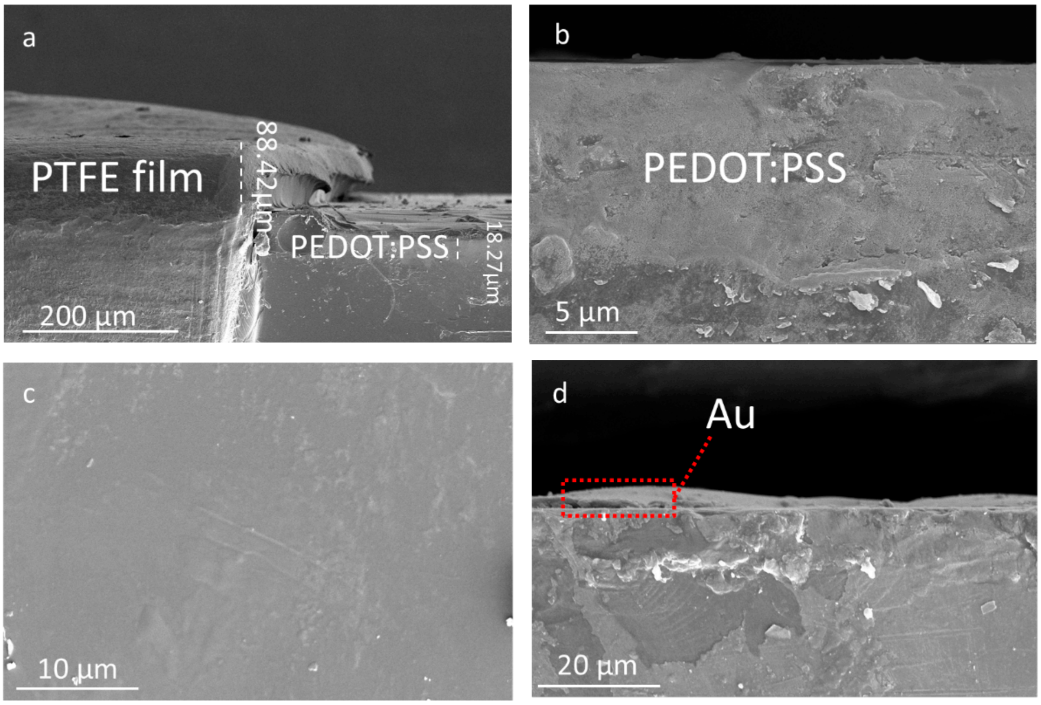

Figure S4. Scanning electron microscopy images of the PEDOT: PSS layer and electrode interface. (a) Cross-sectional view of the PTFE/PEDOT: PSS stack, showing continuous layer contact. (b) High-magnification surface morphology of the PEDOT: PSS film. (c) Surface view of the PEDOT: PSS. (d) Cross-sectional view of the Au electrode. Scale bars are indicated in each panel.

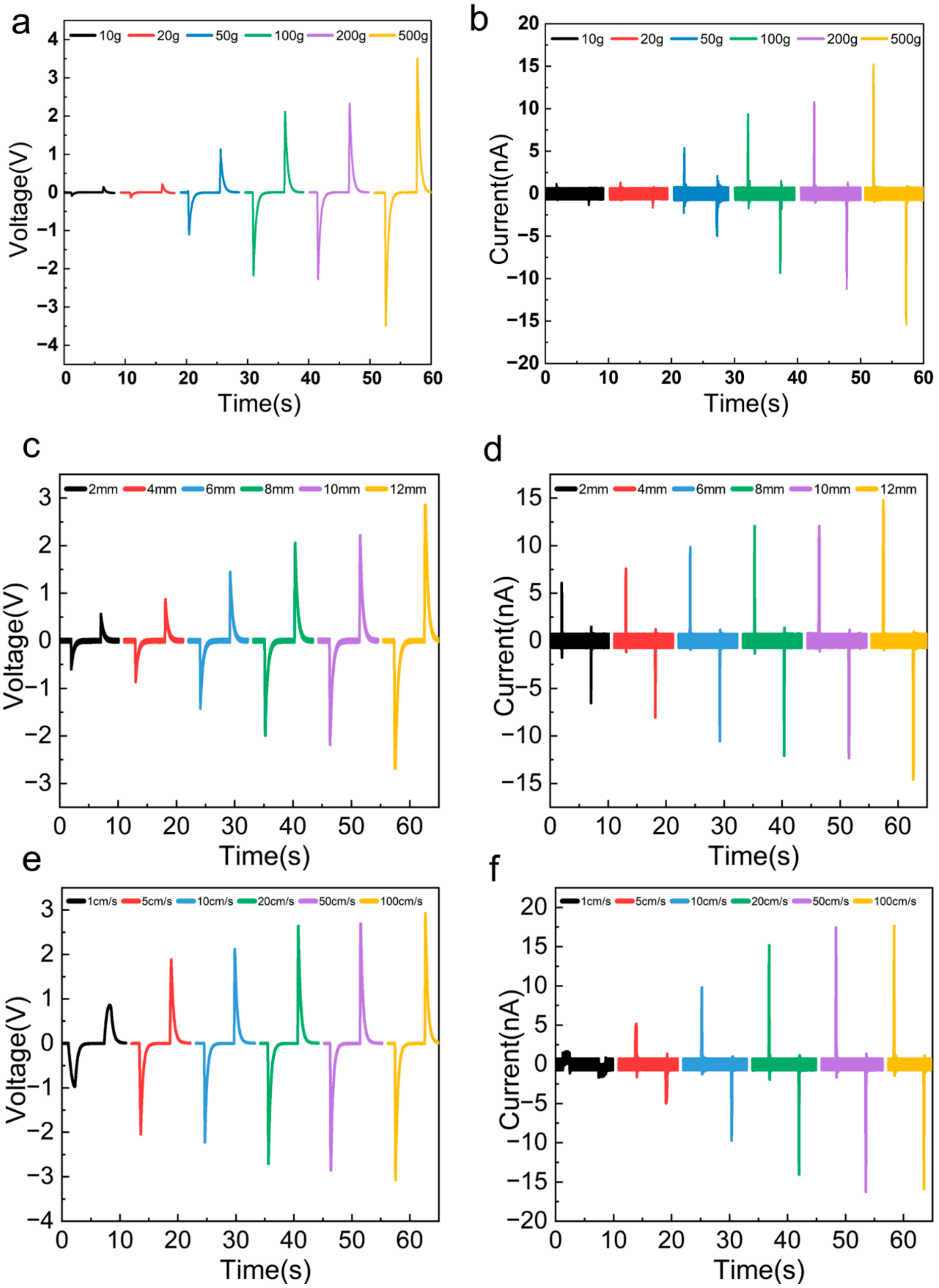

Figure S5. Open-circuit voltage and short-circuit current of the tactile interface sensor measured by an electrometer under varied sliding conditions. (a, b) Open-circuit voltage and short-circuit current under different applied normal pressures. (c, d) Open-circuit voltage and short-circuit current for sensors with different effective widths. (e, f) Open-circuit voltage and short-circuit current under different sliding speeds.

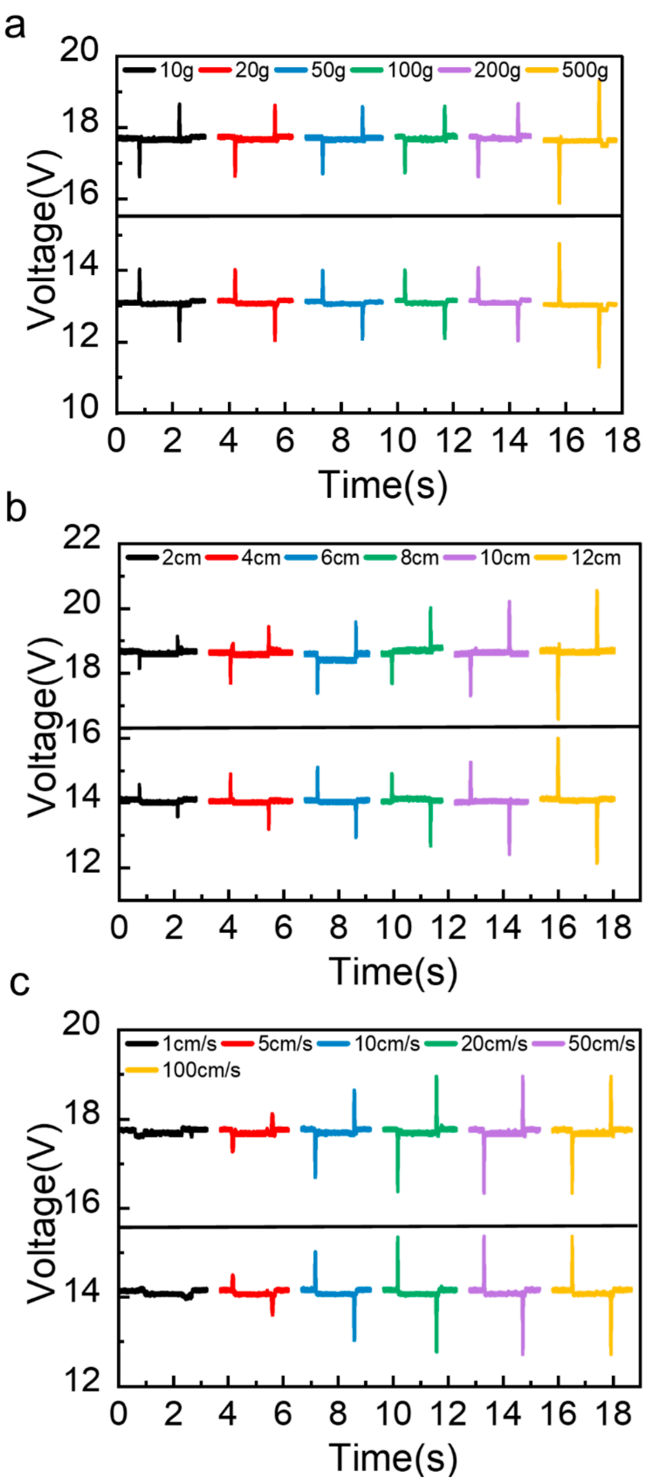

Figure S6. Voltage outputs of the tactile interface sensor measured by an MCU under varying mechanical conditions (baseline not zeroed). (a) Voltage signals under different applied normal pressures. (b) Voltage signals for sensors with different effective widths. (c) Voltage signals under different sliding speeds.

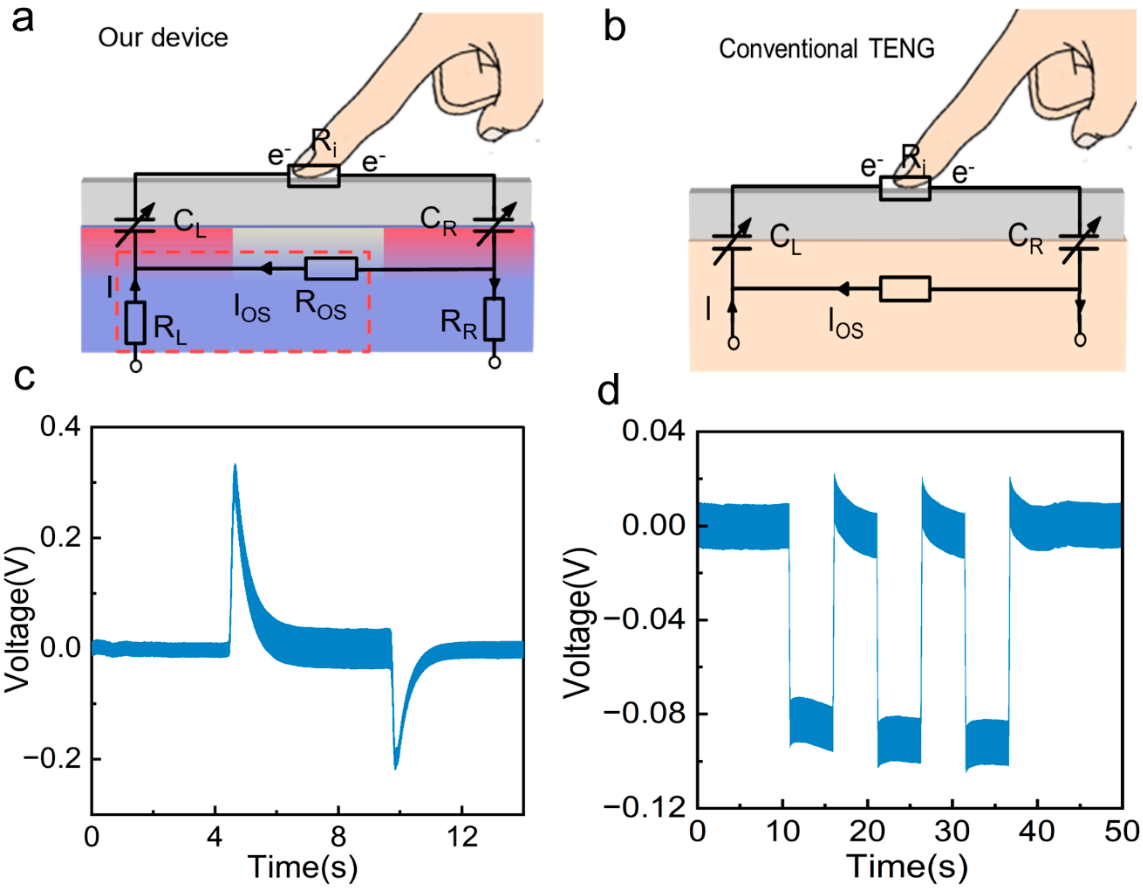

Figure S7. Comparison of equivalent circuit models and electrical outputs between the proposed sliding-gate tactile sensor and a conventional triboelectric nanogenerator. (a) Equivalent circuit model of the proposed sensor. (b) Equivalent circuit model of a conventional triboelectric nanogenerator (same structure but without PEDOT:PSS semiconductive layer). (c) Representative voltage output under a sliding gesture. (d) Representative voltage output of a conventional triboelectric nanogenerator under a sliding gesture.

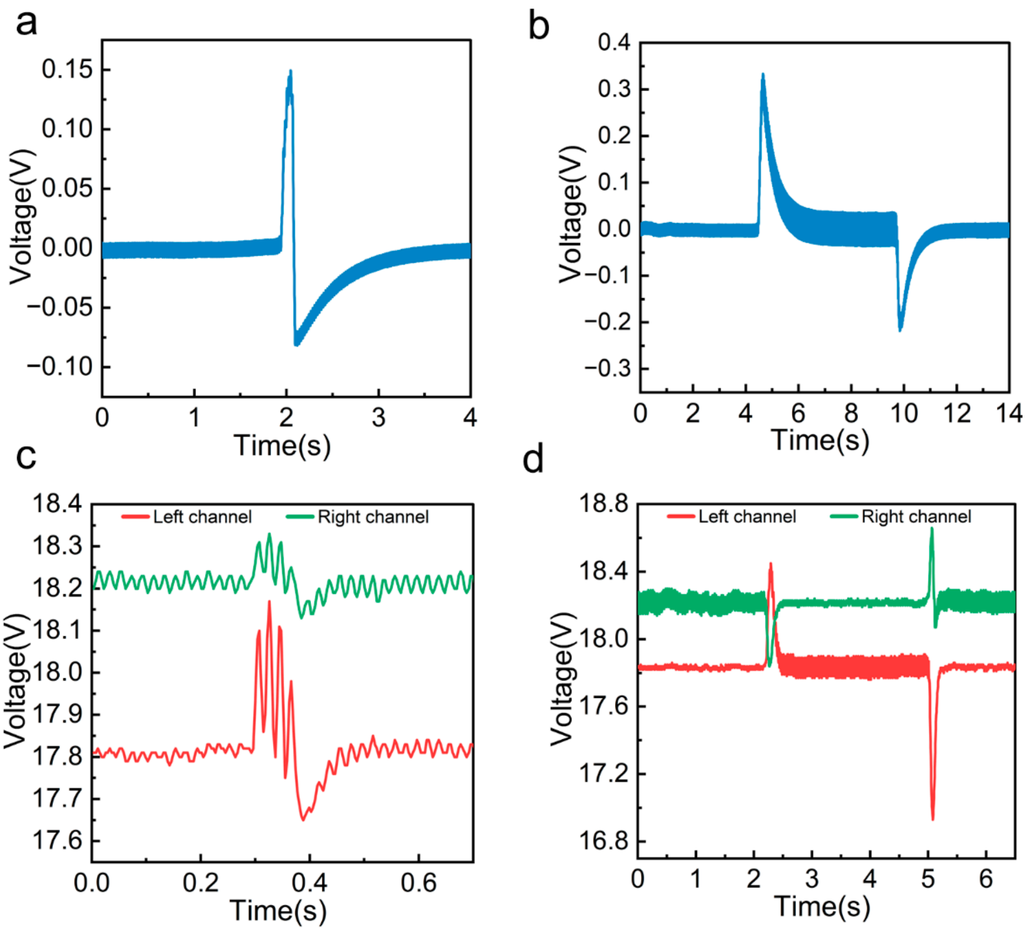

Figure S8. Voltage signals measured by different readout circuits under tapping and sliding gestures. (a) Voltage signal of a tapping gesture measured by a high-impedance electrometer. (b) Voltage signals measured by a high-impedance electrometer under left and right sliding motions. (c) Voltage signal of a tapping gesture measured by an MCU (baseline not zeroed). (d) Voltage signals measured by an MCU under left and right sliding motions (baseline not zeroed).

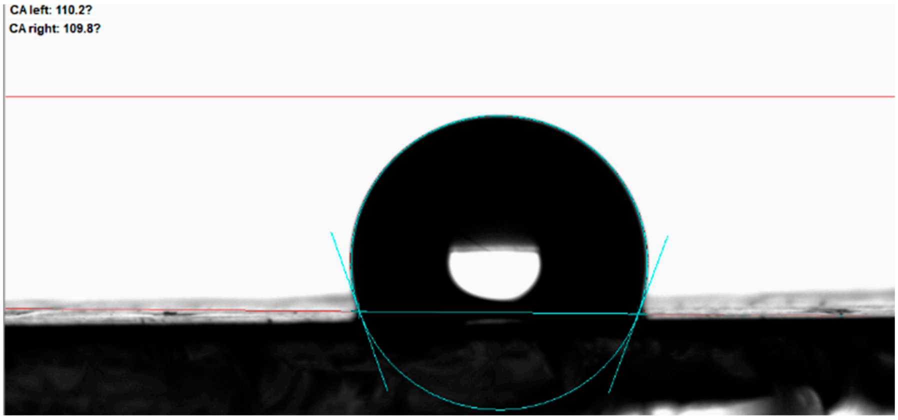

Figure S9. Contact angle of water on PTFE.

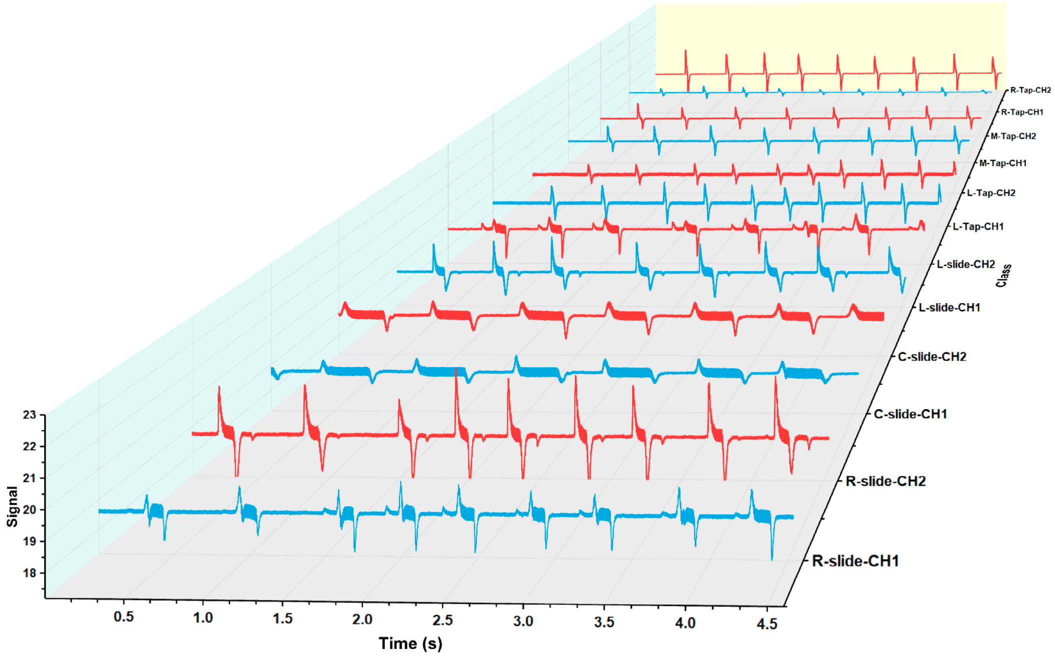

Figure S10. Representative waveform data of six gesture classes.

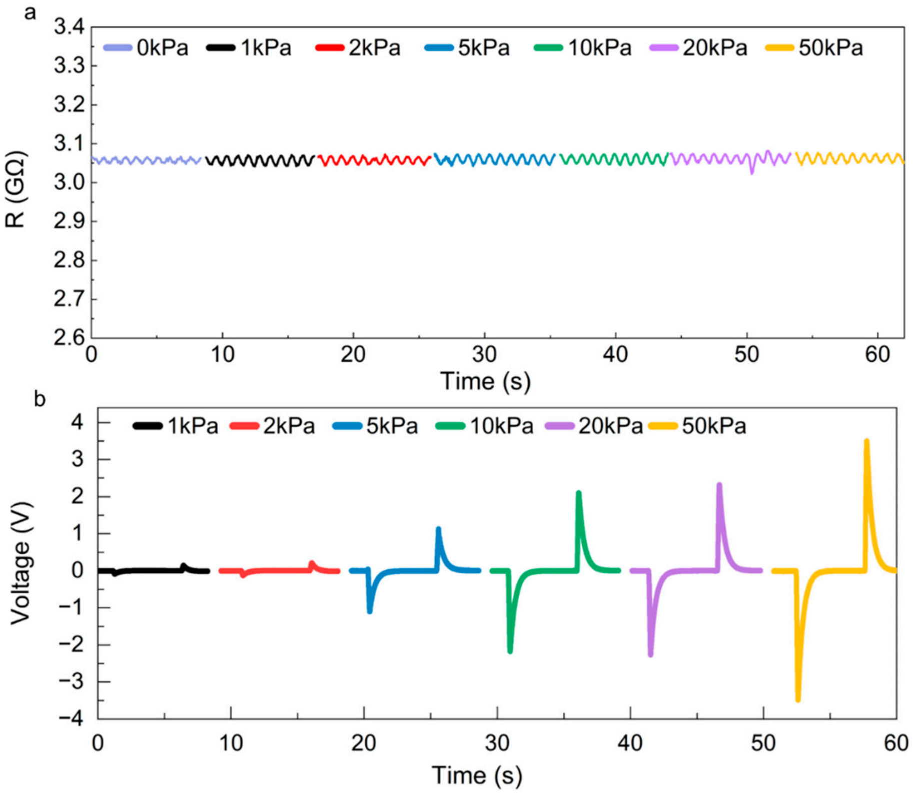

Figure S11. Pressure-dependent resistance variation and corresponding voltage outputs of the sliding-gated tactile interface sensor. (a) Resistance measured under a constant-current bias (1 μA) with a voltage compliance > 210 V at different applied pressures. (b) Representative output voltage waveforms at different applied pressures under the same measurement conditions.

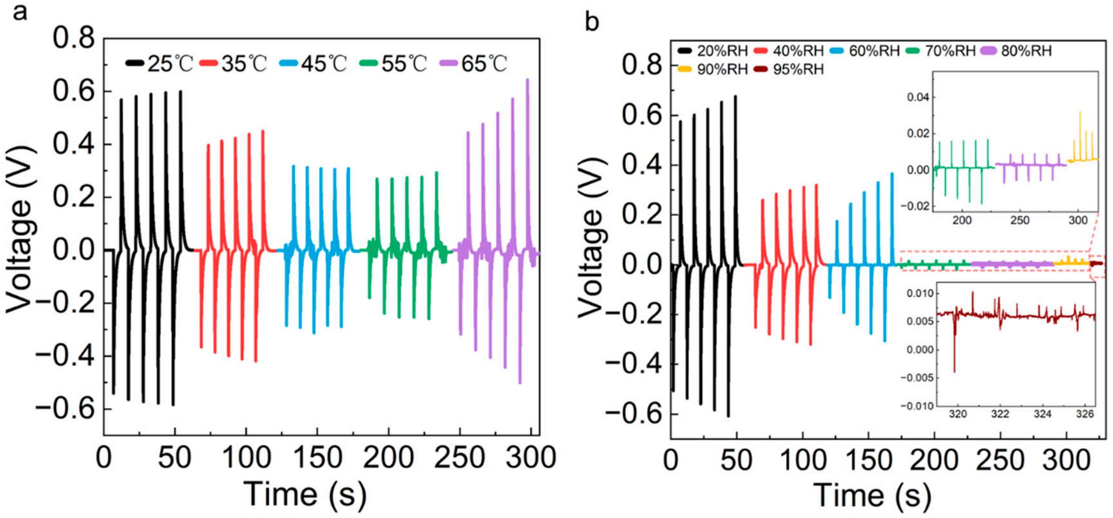

Figure S12. (a) Output under different temperatures; (b) output under different relative humidities.

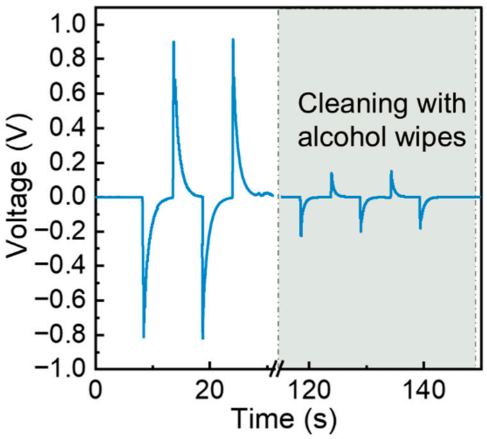

Figure S13. Output before and after cleaning with alcohol wipes.

Table S1. Performance comparison of representative tactile/gesture sensors in terms of power supply, channel configuration, recognition mode, durability, and integration level.

Sensor	Power	Channels	Recognition mode	Direction sensing	Durability	Integration	Output voltage
Sliding-gated device	Self-powered	Dual	Slide/Touch	Yes	>4000 cycles	High	-5-5 V
Piezoelectric device[2]	Self-powered	Array	Slide	No	250 cycles	Low	-0.2-0.2 V
Tribotronic device[3]	Externally biased	Dual	Touch	No	~2400 cycles	High	-
Iontronic pressure device[4]	Externally biased	Array	Static Pressure	No	>5000 cycles	Medium	-
Dynamic-static tactile system[5]	Self-powered	Array	Slide/Touch	Yes	-	Medium	-
Ionic skin[6]	Externally biased	Array	Slide/Touch	No	~2000 cycles	Low	-
Graphene device[7]	Externally biased	Array	Static Pressure	No	>5000 cycles	Low	-
Triboelectric nanogenerator[8]	Self-powered	Single	Slide/Touch	Yes	-	Low	0-200 V

Table S2. Comparison between the proposed sliding-gated tactile interface and representative capacitive side-key solutions used in smartphones.

Device	Sensitivity	Power consumption	Form factor	Manufacturing complexity
Proposed sliding-gated tactile interface (this work)	Sliding direction, speed, click pressure, touch position	Self-powered signal	Small	Simple electrodes; printing/coating; lamination
Commercial capacitive touch side key	Press force	Needs active scanning	Medium	Electrode arrays; printing/shielding; lamination
Commercial pressure-sensitive side key	Press force	Needs active scanning	Medium	Electrode arrays; printing/shielding ; lamination

Table S3. Layer-wise configuration of the CNN+MLP network used for gesture classification.

Layer	Operation	Channels	Kernel size	Stride	Padding	Pooling	Output
0	Input	2→2	-	-	-	-	2X499
1	Conv1D + ReLU	2→16	9	1	4	-	16X499
2	MaxPool1D	-	-	-	-	2	16X249
3	Conv1D + ReLU	16→32	7	1	3	-	32X249
4	MaxPool1D	-	-	-	-	2	32X124
5	Conv1D + ReLU	32→64	5	1	2	-	64X124
6	Global average pooling	64→64	-	-	-	-	64
7	Fully connected + ReLU	64→32	-	-	-	-	32
8	Dropout	-	-	-	-	P=0.3	32
9	Fully connected	32→6	-	-	-	-	6
10	Softmax (inference)	-	-	-	-	-	

References

1. Pan, C.; Cao, L.N.Y.; Meng, J.; Jia, L.; Hu, W.; Wang, Z.L.; Pu, X. Field Effect Nanogenerator Operated by Sliding Gates. *Energy Environ. Sci.* **2024**, *17*, 1132–1140, doi:10.1039/D3EE03496H.
2. Shin, K.; Sim, M.; Choi, E.; Park, H.; Choi, J.-W.; Cho, Y.; Sohn, J.I.; Cha, S.N.; Jang, J.E. Artificial Tactile Sensor Structure for Surface Topography through Sliding. *IEEEASME Trans. Mechatron.* **2018**, *23*, 2638–2649, doi:10.1109/TMECH.2018.2870917.
3. Xue, F.; Chen, L.; Wang, L.; Pang, Y.; Chen, J.; Zhang, C.; Wang, Z.L. MoS_2 Tribotronic Transistor for Smart Tactile Switch. *Adv. Funct. Mater.* **2016**, *26*, 2104–2109, doi:10.1002/adfm.201504485.
4. Bai, N.; Wang, L.; Xue, Y.; Wang, Y.; Hou, X.; Li, G.; Zhang, Y.; Cai, M.; Zhao, L.; Guan, F.; et al. Graded Interlocks for Iontronic Pressure Sensors with High Sensitivity and High Linearity over a Broad Range. *ACS Nano* **2022**, *16*, 4338–4347, doi:10.1021/acsnano.1c10535.
5. Zhao, H.; Qian, W.; Guo, C.; Zhang, Y.; Wang, J.; Dan, H.; Zhang, Y.; Bowen, C.R.; Yang, Y. A Switchable Dynamic-Static Tactile System for Augmented Haptic Secret Communication. *Sci. Adv.* **2025**.
6. Yang, P.-A.; Hu, X.; Li, R.; Lu, Y.; Shou, M.; Sun, R.; He, Y.; Wang, L.; Chen, Q. Dual-Mechanism Layered Ionic Skin: For Quick Touch Position Sensing and High-Precision Touch Intensity Detection. *J. Colloid Interface Sci.* **2026**, *708*, 139778, doi:10.1016/j.jcis.2025.139778.
7. Zhu, B.; Niu, Z.; Wang, H.; Leow, W.R.; Wang, H.; Li, Y.; Zheng, L.; Wei, J.; Huo, F.; Chen, X. Microstructured Graphene Arrays for Highly Sensitive Flexible Tactile Sensors. *Small* **2014**, *10*, 3625–3631, doi:10.1002/smll.201401207.
8. Yin, G.; Liang, X.; Liu, R.; Xu, X.; Zhang, X.; Mo, Y.; Zhou, L.; Wang, S.; Guo, Z.; Liu, Y.; et al. A Single-channel Tactile-slip Triboelectric Nanogenerator for the Intelligent Performance Evaluation of Humanoid Robots. *Adv. Funct. Mater.* **2025**, e19384, doi:10.1002/adfm.202519384.
9. Ming, W.; Zhao, Y.; Zhang, Z.; Qiu, W.; Xu, Y.; Guo, X.; Zhang, G. Self-Powered Handwritten Letter Recognition Based on a Masked Triboelectric Nanogenerator for Intelligent Personal Protective Equipment. ACS Appl. Mater. Interfaces 2024, 16, 57936–57945, doi:10.1021/acsami.4c14677.
10. Zhang, T.; Manshaii, F.; Bowen, C.R.; Zhang, M.; Qian, W.; Hu, C.; Bai, Y.; Huang, Z.; Yang, Y.; Chen, J. A Flexible Pressure Sensor Array for Self-Powered Identity Authentication during Typing. Sci. Adv. 2025, 11, eads2297, doi:10.1126/sciadv.ads2297.
11. Chen, Z.; Jin, Y.; Li, Z.; Wang, B.; Liu, B.; Xu, B.; Gong, F.; Jiang, L.; Li, H. An AI-powered, All-printed, Scalable, Stretchable Triboelectric E-skin for Multifunctional Perception in Dexterous Hand. Adv Funct Materials 2026, e27673, doi:10.1002/adfm.202527673.
12. Han, C.; Cao, Z.; An, Z.; Zhang, Z.; Wang, Z.L.; Wu, Z. Multimodal Finger-Shaped Tactile Sensor for Multi-Directional Force and Material Identification. Adv. Mater. 2025, 37, 2414096. https://doi.org/10.1002/adma.202414096
